# Foetal Radiation Dose and Risk from Diagnostic Radiology Procedures: A Multinational Study

**DOI:** 10.5402/2013/318425

**Published:** 2012-09-25

**Authors:** Ernest K. Osei, Johnson Darko

**Affiliations:** ^1^Department of Medical Physics, Grand River Regional Cancer Center, Kitchener, Canada N2G 1G3; ^2^Department of Physics and Astronomy, University of Waterloo, Waterloo, Canada N2L 3G1; ^3^Cancer Center of Southeastern Ontario, Kingston General Hospital, Kingston, Canada K7L 5P9; ^4^Department of Oncology, Queens University, Kingston, Canada K7L 5P9

## Abstract

In diagnostic radiology examinations there is a benefit that the patient derives from the resulting diagnosis. Given that so many examinations are performed each year, it is inevitable that there will be occasions when an examination(s) may be inadvertently performed on pregnant patients or occasionally it may become clinically necessary to perform an examination(s) on a pregnant patient. In all these circumstances it is necessary to request an estimation of the foetal dose and risk. We initiated a study to investigate fetal doses from different countries. Exposure techniques on 367 foetuses from 414 examinations were collected and investigated. The FetDoseV4 program was used for all dose and risk estimations. The radiation doses received by the 367 foetuses ranges: <0.001–21.9 mGy depending on examination and technique. The associated probability of induced hereditary effect ranges: <1 in 200000000 (5 × 10^−9^) to 1 in 10000 (1 × 10^−4^) and the risk of childhood cancer ranges <1 in 12500000 (8 × 10^−8^) to 1 in 500 (2 × 10^−3^). The data indicates that foetal doses from properly conducted diagnostic radiology examinations will not result in any deterministic effect and a negligible risk of causing radiation induced hereditary effect in the descendants of the unborn child.

## 1. Introduction

In diagnostic radiology examinations, there is a benefit that the patient derives from the resulting diagnosis, provided that they are fully justified. However, given that so many examinations are performed each year, it is inevitable that there will be occasions when an examination will be performed on a woman who subsequently discovers that she was pregnant at the time of her examination(s). It may also occasionally become clinically necessary to perform a radiological examination(s) on a woman who is known to be pregnant. In the later case, there must be rigorous justification of the examination and the procedure itself must be optimized to minimise the foetal dose [1–3]. In order to avoid the former, some special rules have been developed to apply to the exposure of potentially pregnant women (who are or who may be pregnant) in which radiological examinations of such women are restricted to a certain period following menstruation [4–7]. 

For the protection of the foetus from occupational exposure of the pregnant worker, the International Commission on Radiological Protection (ICRP) [1, 2] considers that if a female worker has declared (i.e., notified her employer) that she is pregnant, additional controls have to be considered to protect the embryo/foetus. It is the Commission's policy that the methods of protection at the workplace for women who are pregnant should provide a level of protection for the embryo/foetus, that is, broadly similar to that provided for members of the public. Therefore, the working conditions of a pregnant worker, after declaration of pregnancy, should be such as to ensure that the additional dose to the embryo/foetus would not exceed about 1 mSv during the remainder of the pregnancy [1, 2]. Irrespective of how these protective measures are applied in practice, it is almost inevitable that inadvertent foetal radiation exposures do occur. In these circumstances, it is necessary to consult an expert in medical physics and request that the foetal dose and hence the projected risks be estimated. This may require the medical physicist to calculate the dose based on the knowledge of the technique factors used, or a simulation of the examination using a phantom loaded with dosimeters or review the data from published scientific literature [7–20]. 

Osei and Faulkner [11] estimated the foetal dose of 50 pregnant women in the north of England. Doses to the embryo/foetus varied between less than 0.01 *μ*Gy and 117 mGy, depending on the examination and the gestational ages ranged between 2 and 24 weeks. Angel et al. [18] used Monte Carlo simulations to investigate foetal radiation dose resulting from an abdominal and pelvic examination for a range of patient gestational age and maternal size. The normalized foetal dose estimates from the Monte Carlo simulations ranged from 7.3 to 14.3 mGy/100 mAs, with an average of 10.8 mGy/100 mAs. Doshi et al. [19] also investigated foetal radiation dose from CT pulmonary angiography in late pregnancy. Dose measurements were made on three helical CT scanners, with an anthropomorphic phantom representing the chest and abdomen in late gestation. The estimated foetal doses from the CT scans of the maternal chest were in the range of 60–230 mGy, however by modulating the mA, shielding with a lead coat and scanning 5 cm shorter lengths, foetal doses were reduced by 10%, 35% and 56%, respectively. The Health Protection Agency, UK [20] has recently published foetal doses for some common diagnostic medical exposures. Foetal doses were derived from doses to the uterus observed in recent UK surveys. The foetal doses ranged from about 0.001 mGy to 50 mGy depending on the examination. 

An accurate approach to provide an estimate of foetal dose, either prospectively or retrospectively is to assess the dose for the individual patient using the technique parameters that were used for the patient's examination and taking into account both foetal depth and size. Simply looking up the average uterus dose for a specific examination may lead to underestimation or over estimation of the foetal dose [8–11]. Requests for foetal absorbed dose and associated radiation risk estimates are usually sent to medical physics departments where qualified medical physicists with expertise in foetal dose calculations will estimate the dose and risks associated with the exposure. In this study, we contacted several of these departments in different countries where requests for foetal dose estimate have been sent. Specific values for the following parameters were requested: projection and view (e.g., AP, PA) for each examination, beam quality (e.g., kVp, filtration), source-to-imager receptor distance, machine outputs, and techniques parameters (kVp, tube current, exposure time, etc.) used for the examination(s). In all, we collected and studied exposure data of 367 foetuses and 414 examinations from five institutions in five countries. The purpose of this paper is to present the results of this study.

## 2. Materials and Methods

### 2.1. Foetal Dose Calculations

We contacted several departments in different countries where requests for foetal dose estimate are usually sent. Specific values for the exposure parameters for simple examinations (e.g., chest, etc.), complex (fluoroscopic) examinations (e.g., barium meal, etc.), and CT scans were requested using (see the tables (1A–1C) in Appendix 1 in supplementary material available online at http://dx.doi.org/10.5402/2013/318425). All foetal doses and risks were calculated using the most recent version of FetDose [8] (FetDose V4). FetDose is a computer program developed for the estimation of the radiation dose to the foetus. It calculates the absorbed dose and risk to the foetus from conventional radiography, fluoroscopy, computed tomography, and radiation therapy procedures performed on the pregnant patient. It also calculates the dose to the foetus from occupational exposure of the pregnant worker in the radiological department. The normalized uterus doses (NUD) used for foetal dose calculations are taken from NRPB SR262 [12] and NRPB-SR250 [17]. A more detailed information on the methods used for foetal absorbed dose calculation and hence risk estimations using the FetDose program can be found elsewhere [8], however a short summary is given here. The foetal absorbed dose from a series of radiographic examinations, *D*_*f*_, when the quantity supplied is the entrance surface dose (ESD_rad_) for each radiograph is calculated as [8]

(1) $$\begin{array}{c} D_{f}=\sum_{i=1}^{n}{\text{NUD}}_{d,\text{ESD},i}*{\text{ESD}}_{\text{rad},i}*\text{S}{\text{F}}_{i}, \end{array}$$

where

(2) $$\begin{array}{c} {\text{ESD}}_{\text{rad}}={\left[\left(\frac{\text{FS}{\text{D}}_{\text{QA}}}{\text{FS}{\text{D}}_{\text{EX}}}\right)\left(\frac{\text{kV}{\text{p}}_{\text{EX}}}{\text{kV}{\text{p}}_{\text{QA}}}\right)\right]}^{2}*\text{output}*\text{mAs}. \end{array}$$

*n* is the number of radiographs, NUD_*d*,ESD_ is the uterus dose at a mean foetal depth *d* normalized to free-in-air entrance surface dose, and SF_*i*_ is the foetal size factor (i.e., uterus to foetus dose conversion factor). For complete examinations, including fluoroscopy (e.g., barium enema, barium meal, etc.), involving both spot films and screening procedures of different areas of the body, the total foetal dose is calculated by summing the contributions from both the spot films and screening procedures as

(3) Df=∑i=1nNUDd,ESD,i∗ESDrad,i∗SFi +∑j=1mNUDd,ESD,j∗ESDscreening,j∗SFj,

where

(4) ESDscreening=[(FSDQAFSDEX)(kVpEXkVpQA)]2 ∗output  rate∗mA∗time.

If the quantity provided is the dose-area product per examination, in Gy-cm^2^, then the foetal absorbed dose from a series of radiographic examinations is calculated from

(5) $$\begin{array}{c} D_{f}=\sum_{i=1}^{n}\text{DA}{\text{P}}_{i}*{\text{NUD}}_{d,\text{DAP},i}*\text{S}{\text{F}}_{i}, \end{array}$$

where NUD_*d*,DAP_ is the uterus dose at a mean foetal depth *d* normalized to the dose-area product, and DAP_*i*_ is the dose-area product for each examination, *i*. 

The foetal dose from a series of computed tomography scans is calculated from

(6) $$\begin{array}{c} D_{f}=\frac{\text{NU}{\text{D}}_{V}*\text{CTD}{\text{I}}_{\text{soft}\;\text{tissue}}*\left(\text{mAs}/100\right)\;}{\text{Pitch}}, \end{array}$$

where CTDI_soft  tissue_ (mGy/100 mAs) is the CTDI_air_ to ICRU muscle (CTDI_soft  tissue_ = CTDI_air_∗1.07) used as an approximation to the dose to soft tissue within the body, NUD_V_ is the sum of normalized doses for all 5 mm slabs lying within the scan volume. The National Radiological Protection Board (NRPB) report SR250 [17] provides normalised organ dose data of 23 series of Monte Carlo calculations modelling the conditions of exposure relevant to 27 common models of scanners. Each set of calculation involved 5 mm transverse section (slab) of a mathematical phantom. Estimates of normalised dose to 27 organs or regions of the phantom are calculated for 208 contiguous slabs of the phantom. The contribution of the normalised dose to the uterus is used in FetDose for the estimation of foetal doses. 

### 2.2. Consequence of Foetal Irradiation

The FetDose program presents the consequence of foetal irradiation in four different ways: (1) risks (the probability that a consequence has to be expected), (2) safety (the probability that a consequence is not expected), (3) equivalent number of chest X-rays, and (4) equivalent period of exposure to natural background radiation. In this paper, the consequence is expressed only as the probability that a consequence is expected (risk). Foetal radiation risk (*R*) is calculated as

(7) $$\begin{array}{c} R=D_{f}*\text{RC}, \end{array}$$

where RC is the risk coefficient for the consequence of interest [8]. Radiation effects considered include childhood cancer induction (risk coefficient used is 8.0 × 10^−5^ per mGy [4–6]), hereditary effects (risk coefficient used is 0.5 × 10^−5^ per mGy [4–6]), decline in IQ (risk coefficient used is 25 × 10^−3^ IQ points per mGy [21]) and severe mental retardation (risk coefficient used is 43 × 10^−5^ per mGy [22]). These risk coefficients have been mainly derived from high doses and doserates, and the extrapolation to low doses is far from verified. Nevertheless, most experts accept that the linear no-threshold (LNT) model best fits available data and should remain the foundation of radiation protection.

## 3. Results and Discussion

**Figure 1:**
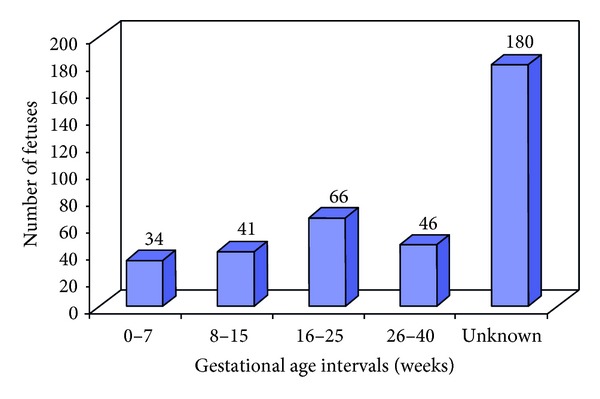
The categorization of the total number of foetuses (367) exposed *in utero* into various gestational age (weeks) intervals.

**Figure 2:**
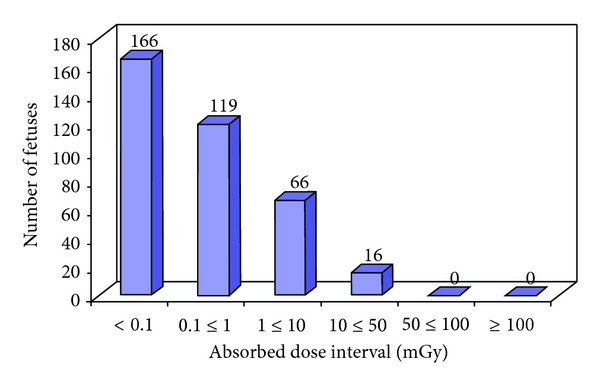
The categorization of the total number of foetus (367) exposed *in utero* into various absorbed dose intervals.

The categorization of the total number of foetuses exposed *in utero* into various gestational age (weeks) intervals is shown in Figure 1.  Figure 2 shows the categorization of the total number of foetus exposed *in utero* into various absorbed dose intervals.  Table 1 shows a summary of the maternal and foetal parameters. Estimated mean and range of entrance surface doses to female adult patients from diagnostic radiology examinations is shown in Table 2. Tables 3 and 4 show a summary of the mean and range of foetal absorbed dose per examination from some simple and special types of diagnostic radiology examinations, respectively. The foetal radiation risks of childhood cancer and hereditary effects per examination are shown in Table 5.  Table 6 also shows the risks of childhood cancer and hereditary effects for the 367 foetuses investigated in this work. 

### 3.1. Foetal Radiation Dose

In this study, exposure parameters from 367 pregnant patients from five institutions in five counties were investigated to estimate the radiation dose and risk to the foetuses. The radiation doses received by the 367 foetuses ranges from less than 0.001 mGy from chest X-ray examination of the pregnant mother to 21.9 mGy from CT of lumbar spine examination (Tables 3 and 4). Some of the women had multiple examinations (2 to 4 different examinations performed on the same patient) and others had repeat examinations (2 to 3 repeated examinations on the same patient), thus contributing to increased foetal doses. According to Osei and Faulkner [11], foetal doses are influenced by the maternal size and foetal depth and size. As a consequence, simply looking up the average uterus dose for a specific examination may lead to underestimation or over estimation of the foetal dose. Foetal dose should therefore be estimated based on the actual technique parameters used for the examination and taking into account the foetal depth and size. However, if the AP thickness of the patient and the foetal depth are not known, an average value of 25 cm and 9 cm, respectively, are good approximations to use [9]. Data in Tables 3 and 4 shows that, for the same examination, the accrual foetal dose can vary significantly from hospital to hospital and from patient to patient depending on several factors including the imaging equipment, technique parameters, patient size, and foetal depth and size. About 78% (285) of the foetus investigated received doses less than 1 mGy, and about 4% (16) of them received radiation doses greater than 10 mGy.

### 3.2. Risks of Death, Malformation, and Mental Retardation

The principal deterministic effects of exposure of the developing embryo or foetus to ionising radiation are death, malformation, growth retardation, and mental impairment. The ICRP [1] has reviewed the risks of harmful tissue reactions and malformations after prenatal irradiation and concluded that no deterministic effects of practical significance would be expected to occur in humans below a dose of at least 100 mGy. The threshold dose [1, 6] for the induction of these deterministic effects following *in utero* exposure of the 367 foetuses all lie very well above the estimated foetal doses, with the maximum foetal dose being 21.9 mGy. There is therefore no risk of death, malformation, growth retardation, or mental impairment from properly conducted diagnostic X-ray examination(s) in this group. In comparison, for the natural course of ordinary pregnancy it is estimated that the general population's total risk of spontaneous abortion, major malformation, mental retardation, and childhood malignancy is about 28.6 percent [23]. According to Brent [24], every healthy woman without personal or family history of reproductive or developmental problems begins her pregnancy with a 1 in 33 (3%) risk for birth defects and 1 in 7 (15%) risk for miscarriage.

### 3.3. Risk of Hereditary Disease

The risk of hereditary effects resulting from exposure of the embryo or foetus is considered to be directly proportional to the radiation dose and fairly independent of the stage of pregnancy after the first three to four weeks of gestation [20]. The probability of induced hereditary effect on the basis of the doses received by the foetuses (Tables 3 and 4) in this study shows that the risk to the individual foetuses ranged from less than 1 in 200000000 (5 × 10^−9^) from chest X-ray examination of the mother to 1 in 10000 (1 × 10^−4^) from lumbar spine CT examination (Table 5). The majority (78%) of the foetuses carry risk less than 1 in 200000 (5 × 10^−6^) (Table 6). The highest foetal dose of 21.9 mGy in this study will carry a risk of hereditary effect of about 1 in 10000 (1 × 10^−4^). The risks values are very low compared to the natural incidence. The natural frequency of genetic disease manifested at birth in human populations has been estimated to be in the range 1 in 100 (1 × 10^−2^) to about 1 in 17 (6 × 10^−2^) if minor congenital abnormalities are included [6, 11, 20]. Thus, the increased genetic risk of 1 in 10000 (1 × 10^−4^) for an individual foetus associated with the highest dose in this study is very small compared with the natural risk of genetic disease. 

### 3.4. Risk of Childhood Cancer

The risk of childhood cancer resulting from exposure of the embryo or foetus is considered to be directly proportional to the radiation dose and fairly independent of the stage of pregnancy after the first three to four weeks of gestation [20]. On the basis of the doses received by the foetuses, the risk of childhood cancer ranges from less than 1 in 12500000 (8 × 10^−8^) to about 1 in 500 (2 × 10^−3^). The risk of 1 in 500 (2 × 10^−3^) associated with the maximum absorbed dose of 21.9 mGy is very comparable to the natural baseline risk of childhood cancer (1 in 500 (2 × 10^−3^)) in the UK [20]. Foetal doses up to about 50 mGy (Table 6) could result in an approximate doubling of the natural baseline risk of childhood cancer. Therefore, those examinations resulting in foetal doses of some tens of mGy during pregnancy should be avoided unless the health of the mother (and indirectly that of the unborn child) would be compromised by delaying the examination until after the birth of the baby [20]. 

## 4. Conclusion

Exposure of the embryo or foetus to high dose ionising radiation can potentially lead to some adverse health effects. However, the radiation dose to the embryo or foetus that is likely to result from any diagnostic procedures should present no risk of causing any deterministic effects such as foetal death, malformation, growth retardation, or mental retardation. Data from this study indicate that foetal doses from properly conducted diagnostic radiology examination will not result in any deterministic effects. Furthermore, the foetal doses presented in this study shows a negligible risk of causing radiation induced hereditary effect in the descendants of the unborn child compared to the natural frequency of genetic disease manifested at birth in human populations. Despite that, all radiological examinations should be clinically justified and the foetal dose kept to a minimum consistent with the diagnostic requirements. It should be noted that doses from some diagnostic radiology examinations may present a very low risk of childhood cancer compared to the natural baseline risk but are certainly not sufficient to justify termination of pregnancy taking into account the health of the mother. It is therefore recommended that [20], any radiological examinations that can result in relatively high foetal doses of some tens of mGy should be avoided on pregnant women, if that can be achieved without serious detrimental effects to their health. 

## Supplementary Material

A study was initiated to investigate foetal doses from different countries. Hence specific values for the following parameters were requested using the tables in Appendix 1: Projection and view (e.g. AP, PA) for each examination, beam quality (e.g. kVp, filtration), source-to-imager receptor distance, machine outputs and techniques parameters (kVp, tube current, exposure time etc) used for the examination(s) performed on the pregnant patient(s). Tables 1A, 1B and 1C were used for conventional radiography (simple) examinations, Fluoroscopic (complex) examinations, and Computed Tomography (complex) examinations respectively.

## Figures and Tables

**Table 1:** Summary of maternal and foetal parameters.

Parameter	Range	Mean
Maternal height (m)	1.4–1.8	1.6
Maternal weight (kg)	40.9–119.8	68.7
Maternal AP thickness (cm)	16.5–31.0	23.7
Maternal age (years)	20–39	30.9
GA (weeks)	2–38	19.2
Foetal depth (cm)*	4.9–9.3	7.1

*If the foetal depth is not provided, a mean value of 9 cm is used [9].

**Table 2:** Estimated mean and range of entrance surface doses to female adult patients from diagnostic radiology examinations.

Projection	Examination	No. of cases	Estimated entrance surface dose (mGy)	
			Range	Mean
AP	Abdomen	56	0.2–10.5	2.62
AP	Chest	38	0.034–0.41	0.12
LAT	Chest	36	0.07–2.51	0.72
PA	Chest	52	0.01–1.62	0.26
PA	Colon	3	4.69–6.24	5.23
	Hip joint	5	0.28–3.13	1.35
AP	Lumbar spine	39	0.4–16.1	4.62
PA	Lumbar spine	4	0.45–0.80	0.56
LAT	Lumbar spine	41	0.29–38.30	12.06
	Lumbosacral joint	30	1.03–28.53	12.24
AP	Pelvis	3	0.59–2.76	1.40
AP	Stomach	16	0.52–6.34	2.50
LAO	Stomach	2	1.59–3.35	2.47
LAT	Stomach	2	2.10–2.60	2.35
LPO	Stomach	6	3.31–3.35	3.33
PA	Stomach	12	0.26–6.34	2.40
AP	Thoracic spine	3	1.25–2.94	2.10
LAT	Thoracic spine	1	3.20	3.20

**Table 3:** The mean and range of foetal absorbed dose per examination from some simple diagnostic radiology examinations.

Projection	Examination	Foetal absorbed dose per examination (mGy)	
		Range	Mean
AP	Abdomen	0.05–3.74	0.80
AP	Chest	<0.001–0.001	0.001
LAT	Chest	<0.001–0.01	0.002
PA	Chest	<0.001–0.01	0.002
PA	Colon	1.66–2.20	1.85
	Hip joint	0.01–0.05	0.03
AP	Lumbar spine	0.11–4.42	1.14
PA	Lumbar spine	0.02–0.80	0.23
LAT	Lumbar spine	0.03–3.89	0.92
	Lumbosacral joint	0.02–0.80	0.25
AP	Pelvis	0.18–1.07	0.49
AP	Stomach	0.01–1.33	0.18
LAO	Stomach	<0.01–0.01	0.01
LAT	Stomach	<0.01–0.01	0.01
LPO	Stomach	<0.01–0.01	0.01
PA	Stomach	<0.01–0.01	0.02
AP	Thoracic spine	<0.001–0.85	0.28
LAT	Thoracic spine	<0.001	<0.001

**Table 4:** The mean and range of foetal absorbed dose per examination from some special types of diagnostic radiology examinations.

Examination	No. of cases	Foetal absorbed dose per examination (mGy)	
		Range	Mean
Barium enema	3	1.14–16.27	7.23
Barium meal	7	0.08–0.19	0.11
Cholangiography	7	1.57–6.65	4.04
Barium follow through	2	0.17–0.64	0.41
IVU	3	0.05–1.33	0.71
KUB	7	0.01–0.59	0.21
Urinary bladder (AP)	1	1.49	1.49
Nephrostomy	2	<0.01–0.37	0.18
CT-abdomen	12	1.04–3.65	1.99
CT-chest	3	0.02	0.02
CT-lumbar spine	10	13.61–21.85	19.05
CT-pelvis	8	1.32–17.06	10.64

**Table 5:** Foetal absorbed dose and risks of childhood cancer and hereditary effects for some common diagnostic radiology examinations.

Projection and examination		Foetal dose range (mGy)	Risk of childhood cancer	Risk of hereditary effects
AP	Abdomen	0.05–3.74	1 in 250000–1 in 3300	1 in 3300000–1 in 50000
AP	Chest	<0.001–0.001	<1 in 12500000–1 in 1250000	<1 in 200000000–1 20000000
LAT	Chest	<0.001–0.01	<1 in 12500000 to 1 in 1250000	<1 in 200000000–1 20000000
PA	Chest	<0.001–0.01	<1 in 12500000 to 1 in 1250000	<1 in 200000000–1 20000000
PA	Colon	1.66-2.20	1 in 10000–1 in 5000	1 in 125000–1 in 100000
	Hip joint	0.01–0.05	1 in 1250000–250000	1 in 20000000–1 in 3300000
AP	Lumbar spine	0.11–4.42	1 in 110000–1 in 2500	1 in 1700000–1 in 50000
PA	Lumbar spine	0.02–0.80	1 in 500000–1 in 17000	1 in 10000000–1 in 250000
LAT	Lumbar spine	0.03–3.89	1 in 500000–1 in 3300	1 in 5000000–1 in 50000
	Lumbosacral joint	0.02–0.80	1 in 500000–1 in 17000	1 in 10000000–1 in 250000
AP	Pelvis	0.18–1.07	1 in 100000–1 in 11000	1 in 1100000–1 in 200000
AP	Stomach	0.01–1.33	1 in 1250000–1 in 10000	1 in 20000000–1 in 140000
LAO	Stomach	<0.01–0.01	<1 in 1250000–1 in 1250000	<1 in 20000000–1 in 20000000
LAT	Stomach	<0.01–0.01	<1 in 1250000–1 in 1250000	<1 in 20000000–1 in 20000000
LPO	Stomach	<0.01–0.01	<1 in 1250000–1 in 1250000	<1 in 20000000–1 in 20000000
PA	Stomach	<0.01–0.01	<1 in 1250000–1 in 1250000	<1 in 20000000–1 in 20000000
AP	Thoracic Spine	<0.001–0.85	1 in 12500000–1 in 14000	<1 in 200000000–1 in 250000
LAT	Thoracic Spine	<0.001	<1 in 12500000	<1 in 200000000

	Barium enema	1.14–16.27	1 in 11000–1 in 1000	1 in 170000–1 in 12500
	Barium meal	0.08–0.19	1 in 170000–1 in 50000	1 in 2500000–1 in 1000000
	Cholangiography	1.57–6.65	1 in 10000–1 in 2000	1 in 125000–1 in 33000
	Barium follow through	0.17–0.64	1 in 100000–1 in 20000	1 in 1100000–1 in 330000
	IVU	0.05–1.33	1 in 250000–1 in 10000	1 in 3300000–1 in 140000
	KUB	0.01–0.59	1 in 1250000– 1 in 20000	1 in 20000000–1 in 330000
AP	Urinary bladder	1.49	1 in 10000	1 in 140000
	Nephrostomy	<0.01–0.37	1 in 1250000–33000	<1 in 20000000–1 in 500000

	CT-Abdomen	1.04–3.65	1 in 12500–1 in 3300	1 in 200000–1 in 50000
	CT-Chest	0.02	1 in 500000	1 in 10000000
	CT-Lumbar spine	13.61–21.85	1 in 1000–1 in 500	1 in 14000–1 in 10000
	CT-Pelvis	1.32–17.06	1 in 10000–1 in 1000	1 in 140000–1 in 11000

**Table 6:** Foetal dose-averaged and risk of childhood cancers and hereditary effects for the 367 foetuses exposed during diagnostic radiology examinations of their mothers.

Foetal dose-averaged	No. of foetuses	Risk of childhood cancer	Risk of hereditary effects
<0.1	166	<1 in 125000	<1 in 2000000
0.1–<1	119	1 in 125000–<1 in 12500	1 in 2000000–<1 in 200000
1–<10	66	1 in 12500–<1250	1 in 200000–<1 in 20000
10–<50	16	1 in 1250–<1 in 250	1 in 20000–<4000
