# Supplementary material for: Foetal Radiation Dose and Risk from Diagnostic Radiology Procedures: A Multinational Study
Source: ISRN Radiol. 2012 Sep 25;2013:318425. doi: 10.5402/2013/318425 (PMC4045527; doi:10.5402/2013/318425)
Supplement: Supplementary file 1 — A study was initiated to investigate foetal doses from different countries. Hence specific values for the following parameters were requested using the tables in Appendix 1: Projection and view (e.g. AP, PA) for each examination, beam quality (e.g. kVp, filtration), source-to-imager receptor distance, machine outputs and techniques parameters (kVp, tube current, exposure time etc) used for the examination(s) performed on the pregnant patient(s). Tables 1A, 1B and 1C were used for conventional radiography (simple) examinations, Fluoroscopic (complex) examinations, and Computed Tomography (complex) examinations respectively. [file 318425.f1.doc]

**Appendix 1A**

Institution City Country

Contact person (Name and email address)…

Conventional radiography (simple) examinations (You may make additional copies of the table)

| # | X-ray tube output | | | Patient information | | | | | Examination parameters | | | | | | | | |
| --- | --- | --- | --- | --- | --- | --- | --- | --- | --- | --- | --- | --- | --- | --- | --- | --- | --- |
|  | Free-in  air output  (mGy/mAs) | FSD  (cm) | TP  (kVp) | GA  (weeks) | Mat. height  (cm) | Mat. weight  (kg) | AP thickness  (cm) | Fetal depth  (cm) | Exams & projections | ESD^*^  (mGy) | DAP  (mGy-cm^2^) | TP  (kVp) | Filtration  (mmAl) | FSD  (cm) | # of films | Field Size | mAs |
|  |  |  |  |  |  |  |  |  |  |  |  |  |  |  |  |  |  |
|  |  |  |  |  |  |  |  |  |  |  |  |  |  |  |  |  |  |
|  |  |  |  |  |  |  |  |  |  |  |  |  |  |  |  |  |  |
|  |  |  |  |  |  |  |  |  |  |  |  |  |  |  |  |  |  |

Please, you may provide as much information as possible,

FSD = Focus-skin-distance TP= tube potential Mat. = Maternal ESD =entrance surface dose (supply if only it was measured)

DAP=dose area product

**Appendix 1B**

Institution City Country

Contact person (Name and email address)…

Fluoroscopic (complex) examinations (You may make additional copies of the table)

| # | X-ray tube output | | | Patient information | | | | | Examination parameters | | | | | | | | |
| --- | --- | --- | --- | --- | --- | --- | --- | --- | --- | --- | --- | --- | --- | --- | --- | --- | --- |
|  | Free-in  air output  (mGy/mAs) | FSD  (cm) | TP  (kVp) | GA  (weeks) | Mat. height  (cm) | Mat. weight  (kg) | AP thickness  (cm) | Fetal depth  (cm) | Exams. Type | Exams & projection | ESD^*^  (mGy) | DAP  (mGy-cm^2^) | TP  (kVpl | Filtration  (mmAl) | FSD  (cm) | Field Size | Time/ current  (sec/mA) |
|  |  |  |  |  |  |  |  |  |  |  |  |  |  |  |  |  |  |
|  |  |  |  |  |  |  |  |  |  |  |  |  |  |  |  |  |  |
|  |  |  |  |  |  |  |  |  |  |  |  |  |  |  |  |  |  |

Please, you may provide as much information as possible,

FSD = Focus-skin-distance TP= tube potential Mat. = Maternal ESD =entrance surface dose (supply if only it was measured)

DAP=dose area product

**Appendix 1C**

CT (complex) examinations (You may make additional copies of the table)

|  | CT Scanner | | | Examination parameters | | | | | | | | |
| --- | --- | --- | --- | --- | --- | --- | --- | --- | --- | --- | --- | --- |
| # | Manu./Model | Axial/  spiral | Multi/  Single slice | Exams type | Slide thickness | # of slices | Couch increment or pitch | mAs per exam | kVp | Rotation time | mAs per rotation | Collimation |
|  |  |  |  |  |  |  |  |  |  |  |  |  |
|  |  |  |  |  |  |  |  |  |  |  |  |  |
|  |  |  |  |  |  |  |  |  |  |  |  |  |
